# Eosinophilic and neutrophilic leukemoid reaction in a woman with spindle cell sarcoma: a case report

**DOI:** 10.1186/1752-1947-4-335

**Published:** 2010-10-21

**Authors:** Michael C Snyder, Carl B Lauter

**Affiliations:** 1 Department of Medicine, William Beaumont Hospital, 3601 W. 13 Mile Road, Royal Oak, MI 48073, USA; 2 Department of Medicine, Divisions of Allergy, Immunology and Infectious Disease, William Beaumont Hospital, 3601 W. 13 Mile Road, Royal Oak, MI 48073, USA

## Abstract

**Introduction:**

We report a case of a patient with marked eosinophilia and neutrophilia as a manifestation of a spindle cell sarcoma.

**Case presentation:**

A 41-year-old African American woman presented with an enlarging, painful mass in her right knee area. Four years previously, she had had a mass similar to this diagnosed as an osteosarcoma, and had undergone a radical resection and hinge-knee replacement. Before the surgery, she was treated with neoadjuvant docetaxel and gemcitabine. A biopsy was taken from the recurrent mass, and histological examination revealed high-grade soft-tissue sarcoma. The patient received no further treatment. Complete blood counts revealed a white blood cell (WBC) count of 13.6 to 17.9 × 10^9^/L, with neutrophils being 8.2 to 10.9 × 10^9^/L and eosinophils 1.8 to 1.9 × 10^9^/L. At readmission six months later, WBC was 126.7 × 10^9^/L, with neutrophils being 57.02 × 10^9^/L and eosinophils 60.82 × 10^9^/L. The eosinophils peaked at 77.79 × 10^9^/L two days later. Evaluations for allergies, infection, and autoimmune mechanisms were negative. Bone marrow revealed increased eosinophils without blasts. After resection, blood counts abruptly decreased to the normal range. Pathology confirmed high-grade spindle cell sarcoma. Approximately one year after resection, the patient was readmitted with metastatic disease to her lungs. During this presentation, her eosinophil and neutrophil count was again increased. WBC was 107.8 × 10^9^/L, with eosinophil count of 47.43 × 10^9^/L and neutrophil count of 44.10 × 10^9^/L. Interleukin-5 was normal, and granulocyte–macrophage colony-stimulating factor (GM-CSF) was elevated at 208.8 (normal < 4.8).

**Conclusion:**

In our case, the patient had eosinophilia and neutrophilia associated with a spindle cell sarcoma, possibly representing a paraneoplastic syndrome secondary to GM-CSF. There were no signs of infectious, allergic, or autoimmune causes for the eosinophilia or neutrophilia. Even though the occurrence of eosinophilia and neutrophilia with malignancy is rare, patients who have either condition without an apparent cause should be checked for malignancy.

## Introduction

Eosinophilia can be a manifestation of a variety of causes, such as infections, drug reactions, allergic reactions, and autoimmune processes. It has also been described in relation to neoplasm as demonstrated by Isaacson and Rapoport in 1941, who presented 34 cases of neoplasm associated with eosinophilia [1]. Since then, there has been an accepted association of eosinophilia with hematologic malignancies such as lymphoma and leukemia. Eosinophilia has also been noted in a myriad of primary tumors such as cancer of the liver, breast, uterus, and ovaries [2]. The association of eosinophilia and sarcomas, however, is rare. Neutrophilic leukemoid reactions, which are more common than eosinophilia, can also be difficult to diagnose with differentials including common etiologies such as infections and solid malignancies, and uncommon causes such as chronic neutrophilic leukemia [3].

Sarcomas are very rare malignancies, making up approximately 1% of adult malignancies and 15% of pediatric malignancies. They can occur at any site of the body, and arise primarily from mesenchymal structures [4]. Osteosarcoma is the most common type of sarcoma of the bone. Spindle cell sarcomas are very closely related to osteosarcomas, except that ostioid is not produced in spindle cell sarcomas. They are treated in a similar fashion.

We present a case of eosinophilia and neutrophilia associated with spindle cell sarcoma probably representing a paraneoplastic syndrome. There were no signs of infectious, allergic, or autoimmune causes for the patient’s eosinophilia or neutrophilia.

## Case presentation

A 41-year-old African American woman presented with an enlarging, painful mass in the right knee area. She had a history of osteosarcoma in her right leg diagnosed four years previously, and had undergone radical resection and hinge-knee replacement at another hospital. She had also been treated with neoadjuvant docetaxol and gemcitabine before her resection.

Owing to her history of osteosarcoma, we took a biopsy from the mass, which was diagnosed histologically as a recurrent, high-grade soft tissue sarcoma. Laboratory investigations revelaed white blood cell (WBC) count of 13.6 to 17.9 × 10^9^/L, neutrophils 8.2 to 10.9 × 10^9^/L, and eosinophils 1.8 to 1.9 × 10^9^/L (reference range WBC 3.3 to 10.7 × 10^9^/L, neutrophils 1.6 to 7.2 × 10^9^/L, eosinophils 0.0 to 0.5 × 10^9^/L). The patient received no further treatment and was discharged.

**Figure 1:**
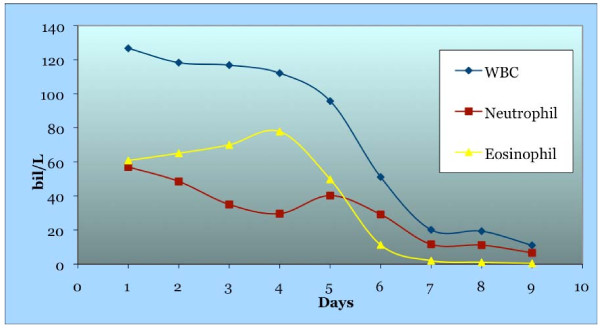
The number of white blood cells (WBC), eosinophils, and neutrophils before and after surgery

Our patient returned six months later with increased pain in her right leg, and marked growth of the leg mass. She also reported a decreased range of motion secondary to the right knee mass. On physical examination, we found a large mass in the right knee area approximately 150 × 200 mm in size, which was hard, fixed and tender. Owing to the size, there was markedly decreased range of motion. There was no pedal edema, and peripheral pulses were intact. There was also an enlarged (15 × 20 mm), hard, slightly tender lymph node in the right inguinal area. There was no rash. There was no loss of sensation distal to the mass. Laboratory investigations revealed WBC 126.7 × 10^9^/L, neutrophils 57.02 × 10^9^/L, and eosinophils 60.82 × 10^9^/L. The eosinophil level peaked two days later at 77.79 × 10^9^/L (Table 1, Figure 1).

**Table 1:** Complete blood count ( × 10^9^/L)

Hospital day	WBC	Neutrophils	Lymphocytes	Monocytes	Eosinophils
1	126.7	57.02	2.53	6.34	60.82

2	118.3	48.5	1.18	3.55	65.06

3	116.8	35	2.1	4	70

4	112.1	29.61	2.9	5.22	77.79

5	95.7	40.19	3.83	1.91	49.76

6	51.1	29.13	5.62	3.58	11.24

7	20.1	11.6	3.6	2.4	2

8	19.3	11.1	4.3	2.6	1

9	11	6.6	2.8	1.1	0.4

WBC = white blood cells.

Owing to her massive eosinophilia and neutrophilia, our patient underwent bone marrow biopsy to exclude leukemia and demonstrate a lack of myeloid clonality. The bone marrow revealed eosinophilia and neutrophilia without increased blast cells. Testing for JAK2 mutation was negative as was flow cytometry. Other investigations included: IgE (3 IU/mL), C3 (174 mg/dL), C4 (35 mg/dL), haemolytic complement (CH) (276 units) (normal ranges 0 to 120 IU/mL, 65 to 190 mg/dL, 15 to 50 mg/dL, 100 to 300 units, respectively). Testing for anti-neutrophil cytoplasmic antibody was negative. Our patient then underwent amputation of her right leg mass, and lymph node excision of her palpable inguinal lymph nodes. Results showed high-grade spindle cell sarcoma with metastasis in three of six lymph nodes. Five days after surgery, our patient’s blood counts returned essentially to normal (WBC 11.0 × 10^9^/L, neutrophils 6.6 × 10^9^/L, and eosinophils 0.4 × 10^9^/L).

Approximately one year later, our patient returned to the hospital for a recurrence of her previously resected osteosarcoma. At this time, she had metastatic disease to the lungs and once again had elevated blood counts: WBC 107.8 × 10^9^/L, neutrophils 49.59 × 10^9^/L and eosinophils 47.43 × 10^9^/L. Interleukin (IL)-5 was 5.0 pg/mL (normal < 10). Granulocyte–macrophage colony-stimulating factor (GM-CSF was elevated at 208.8 pg/mL (normal < 4.8). Our patient decided to have no further intervention for her metastatic disease and to receive palliative care only.

## Discussion

Eosinophilia is defined as mild (350 to 1,500 cells/μL), moderate (1,500 to 5000 cells/uL), or severe (>5,000 cells/μL). It can occur in several disorders such as drug reactions, allergic diseases, and infections. It has also been shown to occur in malignancies, with most occurring in hematologic cases [2]. In 1946, 34 cases of eosinophilia associated with neoplasm were presented by Isaacson and Rapoport [2]. Since then, there have been numerous reports of eosinophilia associated with hematologic cancers and primary neoplasm of the breast, liver, uterus and ovaries [2,5,6]. Neutrophilic leukemoid reactions have also been documented with malignancies [3]. However, the association of eosinophilia with sarcomas is very rare, with only a few cases reported in the literature. Eosinophilia has been reported in two cardiac rhabdomyosarcomas, a chest wall sarcoma, an alveolar sarcoma, an undifferentiated embryonal sarcoma, and two uterine leiomyosarcomas (Table 2)[5-12]. To the best of our knowledge, it has never been reported in the literature with a spindle cell sarcoma.

**Table 2:** Reported cases of eosinophilia associated with sarcomas

Location/Type of Sarcoma	Author	Year
Alveolar soft-part sarcoma	Almansori, et al.	2005

Angiosarcoma	Zeitouni, et al.	2002

Cardiac Rhabdomyosarcoma	Sullivan, et al.	1983

Cardiac Rhabdomyosarcoma	Lo Re III, et al.	2003

Chest wall sarcoma	Hussain, et al.	1994

Embryonal sarcoma	O'Sullivan, et al.	2001

Uterine Leiomyosarcoma	Buka, et al.	1965

Uterine Leiomyosarcoma	Ral, et al.	2003

Uterine Leiomyosarcoma	Onishi, et al.	2005

A number of hypotheses have been put forward regarding the etiology for eosinophilia associated with malignancy. These include the release of protein material from necrosis of the tumor causing an eosinophilotactic response, the release of chemotactic factors for eosinophils from tumor cells, the seeding of metastatic tumor cells to the bone marrow causing production of eosinophils, and the stimulation of bone marrow cells to produce eosinophils by eosinophilotactic factors produced by the tumor cells [13]. It is not know if eosinophilia is a good or poor prognostic sign, as eosinophilia with neoplasm has been associated with both positive and negative prognostic significance [14]. Peripheral eosinophilia has been associated with a worse prognostic sign than tissue eosinophilia, which could have a better prognosis [9].

Eosinophils are produced from pluripotent stem cells in the bone marrow guided through the eosinophil lineage by cytokines and growth factors. There are several cytokines and growth factors that can be involved in the production of eosinophils, with the main cytokines being IL-3 and IL-5, and the main growth factor being GM-CSF [15]. These factors have been shown to induce eosinophil production *in vitro*. IL-3 and GM-CSF have activity on other cells as well, but IL-5 is more specific for eosinophils [15]. These products have also been shown to increase in relation to malignancies with peripheral eosinophilia. Because GM-CSF is not specific for eosinophils, it can also induce production of other cell lines, causing neutrophilic leukemoid reactions.

## Conclusion

Our patient had eosinophilia and neutrophilia associated with a spindle cell sarcoma, possibly representing a paraneoplastic syndrome secondary to GM-CSF. There were no signs of infectious, allergic, or autoimmune causes for the eosinophilia or neutrophilia. Even though the occurrence of eosinophilia and neutrophilia with malignancy is rare, patients who have either with no apparent cause should be screened for malignancy.

## Consent

Written informed consent for publication could not be obtained from the patient as the patient is died and the next-of-kin could not be contacted despite all reasonable attempts. However, every effort has been made to protect patient anonymity and there is no reason to think that the patient or family would object to publication.

## Competing interests

The authors declare that they have no competing interests.

## Authors' contributions

CL analyzed and interpreted patient data. MS wrote the case report. Both CL and MS performed literature search and read/approved final manuscript.
